# Intermediates in monensin biosynthesis: A late step in biosynthesis of the polyether ionophore monensin is crucial for the integrity of cation binding

**DOI:** 10.3762/bjoc.10.34

**Published:** 2014-02-10

**Authors:** Wolfgang Hüttel, Jonathan B Spencer, Peter F Leadlay

**Affiliations:** 1 Department of Biochemistry, University of Cambridge, 80 Tennis Court Road, Cambridge CB2 1GA, UK, Fax: (+44)1223-766002; 2 Institute for Pharmaceutical Sciences, Universität Freiburg, Albertstr. 25, 79104 Freiburg, Germany; 3 Department of Chemistry, University of Cambridge, Lensfield Road, Cambridge CB2 1QW, UK

**Keywords:** antibiotics, biosynthesis, natural products, polyketides, *Streptomyces*, synthetic biology

## Abstract

Polyether antibiotics such as monensin are biosynthesised via a cascade of directed ring expansions operating on a putative polyepoxide precursor. The resulting structures containing fused cyclic ethers and a lipophilic backbone can form strong ionophoric complexes with certain metal cations. In this work, we demonstrate for monensin biosynthesis that, as well as ether formation, a late-stage hydroxylation step is crucial for the correct formation of the sodium monensin complex. We have investigated the last two steps in monensin biosynthesis, namely hydroxylation catalysed by the P450 monooxygenase MonD and *O*-methylation catalysed by the methyl-transferase MonE. The corresponding genes were deleted in-frame in a monensin-overproducing strain of *Streptomyces cinnamonensis*. The mutants produced the expected monensin derivatives in excellent yields (ΔmonD: 1.13 g L^−1^ dehydroxymonensin; ΔmonE: 0.50 g L^−1^ demethylmonensin; and double mutant ΔmonDΔmonE: 0.34 g L^−1^ dehydroxydemethylmonensin). Single crystals were obtained from purified fractions of dehydroxymonensin and demethylmonensin. X-ray structure analysis revealed that the conformation of sodium dimethylmonensin is very similar to that of sodium monensin. In contrast, the coordination of the sodium ion is significantly different in the sodium dehydroxymonensin complex. This shows that the final constitution of the sodium monensin complex requires this tailoring step as well as polyether formation.

## Introduction

**Scheme 1:**
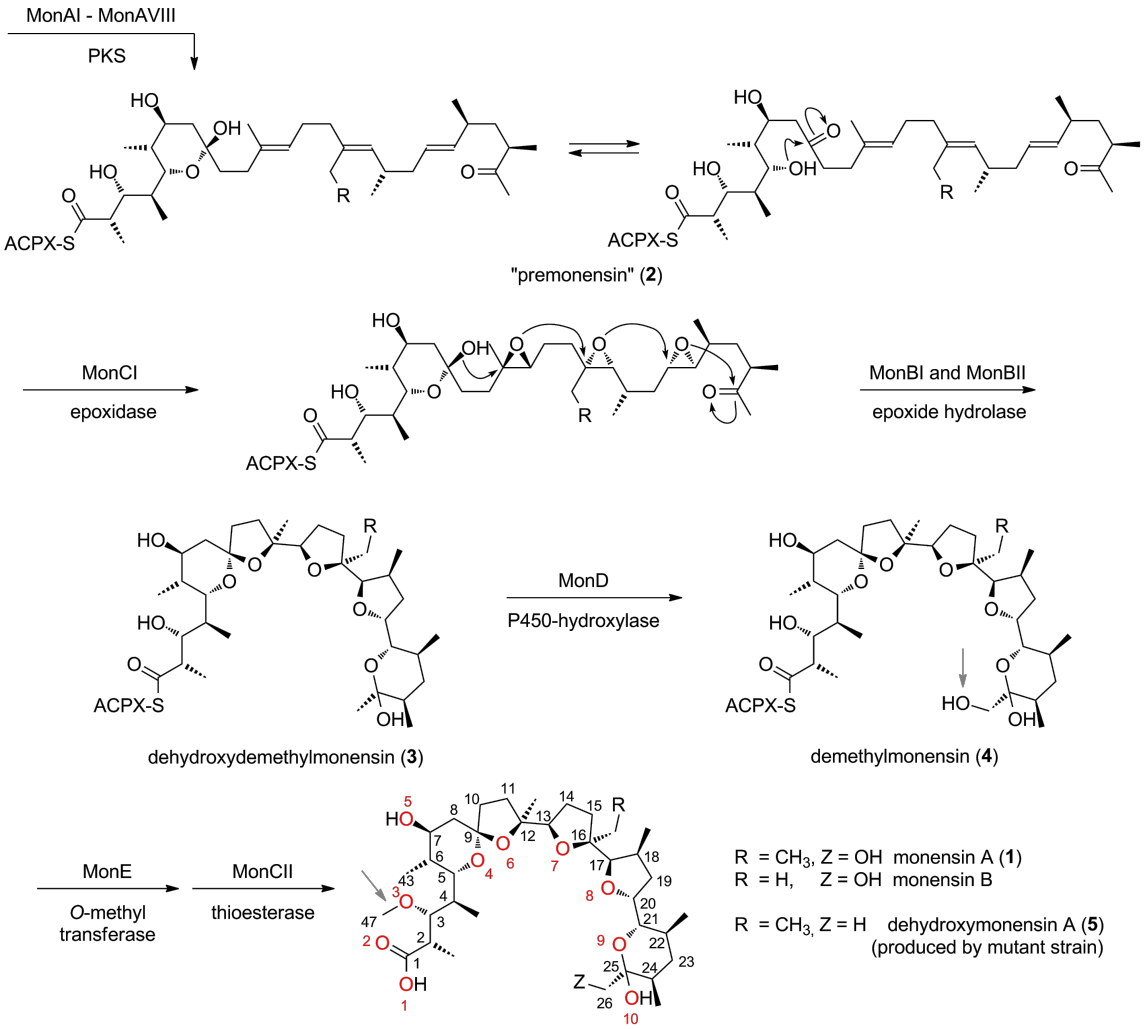
The proposed pathway for monensin biosynthesis in *Streptomyces cinnamonensis*. The polyketide synthase (PKS) initially produces an enzyme-bound triene, which is transferred to a discrete acylcarrier protein (ACPX) to give **2**. After oxidative cyclisation via **3** and **4** the ACP-bound product is finally hydrolysed by MonCII to the free monensin A (**1**, R = CH_3_, Z = OH). Monensin B is a minor product of fermentation. For the final product the atom numbering of the central carbon chain and oxygen atoms (red) is shown.

Monensin A (**1**) from *Streptomyces cinnamonensis* is one of the most prominent and best-studied of the polyether class of complex polyketides, an important and extensive group of natural products including a large number of antibiotic ionophores [1–5] as well as remarkable marine toxins such as the brevetoxins [6–7]. The antibiotic polyethers adopt a characteristic conformation in which multiple oxygen atoms provide ligands for a centrally-held specific cation (sodium in the case of monensin A) while the external surface is exclusively non-polar. This confers the ability to transport the cation across cell membranes leading to dissipation of the membrane potential and cell death. The toxicity of polyethers has limited their clinical use except in animal husbandry, but they are attracting renewed interest for their antimalarial [8–9], antiviral [10–11] and novel anticancer [12–13] activities. In turn, this has given impetus to current attempts to engineer polyether biosynthetic pathways to provide novel analogues with potentially improved therapeutic properties. We report here fresh insight into the order of the final steps of monensin biosynthesis, facilitated by genetic manipulation of an industrial strain of *S. cinnamonensis*. Further, the crystal structures of two of the monensin analogues obtained reveal the structural basis for the key role of late-stage hydroxylation at C-26 of the monensin molecule. Like other polyether ionophores, monensin is assembled by the polyketide biosynthetic pathway on a modular polyketide synthase (PKS) multienzyme [14]. A model has been proposed [14] for monensin biosynthesis in which an initially-formed (*E*,*E*,*E*)-triene undergoes stereospecific epoxidation to a tri-epoxide and subsequent ring-opening and cyclisation to generate the polyether rings. This model has been confirmed and extended (Scheme 1) by the results of more recent work in which specific genes have been disrupted or deleted in *S. cinnamonensis* [15–20], but much remains to be learned about the timing of the various steps of oxidative ring formation (and hence the true nature of the enzyme-bound intermediates) and about the exact role of the enzymes involved in these late steps. As shown in Scheme 1, the monensin PKS (MonAI-MonAVIII) assembles the carbon skeleton of monensin A from five acetate, one butyrate and seven propionate units. The butyrate unit may be substituted by a further propionate unit, producing monensin B, which bears a methyl instead of an ethyl group at C-16.

It appears that oxidative cyclisation is not initiated before the full-length chain is produced, and that the initial product of the PKS is a linear enzyme-bound (*E*,*E,E)*-triene, “premonensin” (**2**) [19]. The monensin PKS does not have a conventional C-terminal thioesterase domain that would catalyse polyketide chain release, and instead a transferase (MonKSX) transfers the chain to a discrete acyl carrier protein (MonACPX) [20], both of these proteins being encoded within the monensin gene cluster [16]. The flavin-dependent epoxidase MonCI then catalyses three stereospecific epoxidations to give the tri-epoxide **3**, which then undergoes a cascade of ring opening/closing catalysed by the combined action of the unusual epoxide hydrolases MonBI and MonBII, to give the putative protein-bound intermediate dehydroxydemethylmonensin. The next steps, catalysed respectively by the cytochrome P450 hydroxylase MonD and the methyltransferase MonE, are hydroxylation at C-26 and O-methylation of the hydroxy group at C-3. Although hydroxylation is shown as preceding methylation in Scheme 1, the preferred order of these events has not been established. The final catalysed step in biosynthesis is the release of mature monensins A and B from the MonACPX, catalysed by the unusual thioesterase MonCII [20].

The evidently tight coupling between PKS-mediated chain assembly and oxidative cyclisation has hampered efforts to unravel the exact sequence and mechanism of events in the late stages of the biosynthesis. Indirect but suggestive evidence for the formation of the presumed tri-epoxide species has been obtained from the analysis of mutants blocked in either or both of the *monBI* and *monBII* genes [17]. These species, after HPLC isolation, were shown to be chemically competent to be isomerised into authentic monensins by the action of dilute acid. Further progress has been hampered by the instability of the various epoxide species as well as by the complexity of the product mixtures, containing both monensin A-related and monensin B-related compounds. Nevertheless, the structural characterisation of one of the products of such blocked mutants as an epimer of monensin A at C-9 [17] has provided clear evidence that both MonD and MonE are capable of acting on altered substrates, which is encouraging for future engineering experiments aimed at production of novel derivatives.

To help to provide a platform for such experiments, and to further elucidate the timing of catalysis of the post-PKS steps in monensin biosynthesis, we have undertaken the specific deletion of *monD* and *monE*, both individually and in combination, and have structurally characterised the principal compounds produced in each case. We report here that the hydroxylation at C-26 has a decisive effect on the three-dimensional structure of the ionophore-cation complex, as revealed by X-ray crystal structure analysis of both dehydroxymonensin A and demethylmonensin A, each in complex with a sodium ion.

## Results and Discussion

**Figure 1:**
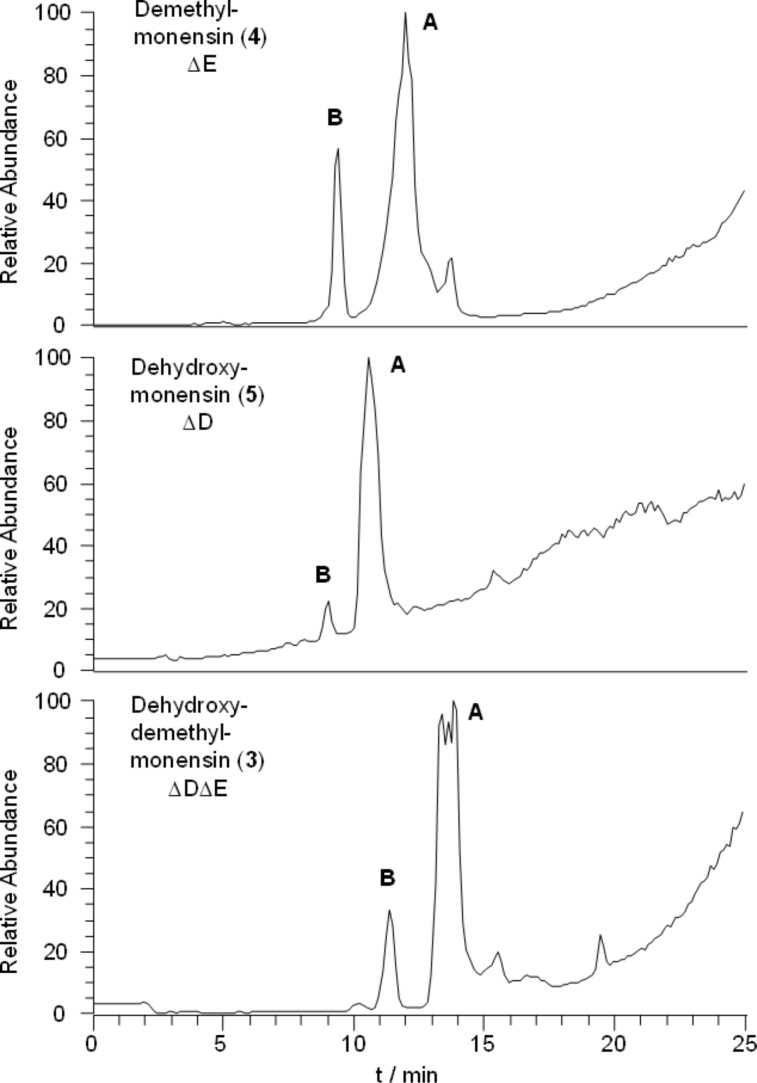
LC–MS-analysis of purified monensin-related metabolites. Monensin B derivatives (peaks marked with B) were identified as minor components of the product mixture. The triple peak for dehydroxydemethylmonensin A (**3**) is due to an enrichment of disodium-species at the start and the end of the fraction. The chromatograms are uncorrected.

PCR-targetted in-frame deletions using the ReDirect [21] version of the RedET recombineering technology [22] were designed to create specific *monD* and *monE* mutants, and also a double mutant ΔmonD ΔmonE (see Experimental). Genetic manipulations were carried out in an industrial monensin-producing strain of *S. cinnamonensis* [19] which routinely produced 3–4 g L^−1^ of product in unoptimised 50 mL shake flask cultures, over 100-fold more than the wild type strain. This overproduction greatly facilitated the characterisation of the products of the ΔmonD and ΔmonE mutants. For polyether production the mutant strains were initially cultivated in the SM-16 medium used for the *S. cinnamonensis* wild type strain [16]. However, in this medium the mutants gave highly variable amounts of products, as judged by LC–MS (data not shown). Therefore, a production medium containing large amounts of oil was used, and a short silica column was used to pretreat the initial extract to remove the large excess of oil before HPLC purification. Analysis by LC–MS revealed that the predicted monensin-related metabolites were indeed produced at elevated levels (see typical results in Figure 1 and Table 1) compared to monensin production in the wild type strain, on either SM-16 or the oil-based medium. The yield of isolated dehydroxydemethylmonensin (**3**) from the double mutant is an order of magnitude lower than the production of monensin from the parent industrial strain, but clearly this precursor, as its ACPX-thioester, is accepted as a substrate by the chain-releasing thioesterase MonCII. The results from the single mutants show that **3** is also a substrate for both MonD and MonE. In the ΔmonD strain substantial amounts of dehydroxydemethylmonensin (**3**) remain unconverted by MonE to dehydroxymonensin (**5**). In contrast, compound **3** was not detected in crude extracts of the ΔmonE strain. These results lend support to the idea that the preferred order of events is hydroxylation at C-26 catalysed by MonD, followed by *O*-methylation at the hydroxy group borne at C-3 catalysed by MonE, as shown in Scheme 1. The amount of dehydroxymonensin A isolated from the MonE mutant was substantially higher than the amount of demethylmonensins obtained from the MonD mutant (Table 1). This can be rationalised if the activity of MonCII is more strongly affected by the lack of a methoxy group at C-3 than by lack of the distal hydroxy group at C-26.

**Table 1:** Monensin derivatives isolated from 250 mL culture medium of *S. cinnamonensis* deletion mutants.

Mutant	Compound^a^	Amount^b^	Crystals

*monD*	dehydroxymonensin (**5**)	284 mg	yes
	dehydroxydemethylmonensin (**3**)	46 mg	no
*monE*	demethylmonensin (**4**)	125 mg	yes
*monD*+ *monE*	dehydroxydemethylmonensin (**3**)	86 mg	no

^a^All products were obtained either as a colourless oil or a colourless solid. ^b^For comparison: 750–1000 mg monensin per 250 mL culture of *S. cinnamonensis* A519 were detected in crude extracts by using the vanillin colorimetric assay [19].

Monensin A-related species were predominant in the product mixtures obtained, with monensin B-related compounds produced only in small amounts (Figure 1). This was not unexpected as the parent strain A519 had been selected and optimised for monensin A production [19]. The spectroscopic properties of the purified 3-*O*-demethylmonensin A were in complete agreement with those previously reported for this compound, isolated as a minor component of a wild-type *S. cinnamonensis* strain [23]. Similarly, the spectroscopic properties of the isolated 26-dehydroxymonensin A were identical with those described for this compound obtained either from a specifically-blocked mutant of wild-type *S. cinnamonensis* [24] or from a wild-type fermentation conducted in the presence of the potent methyltransferase inhibitor metapyrone [25].

**Figure 2:**
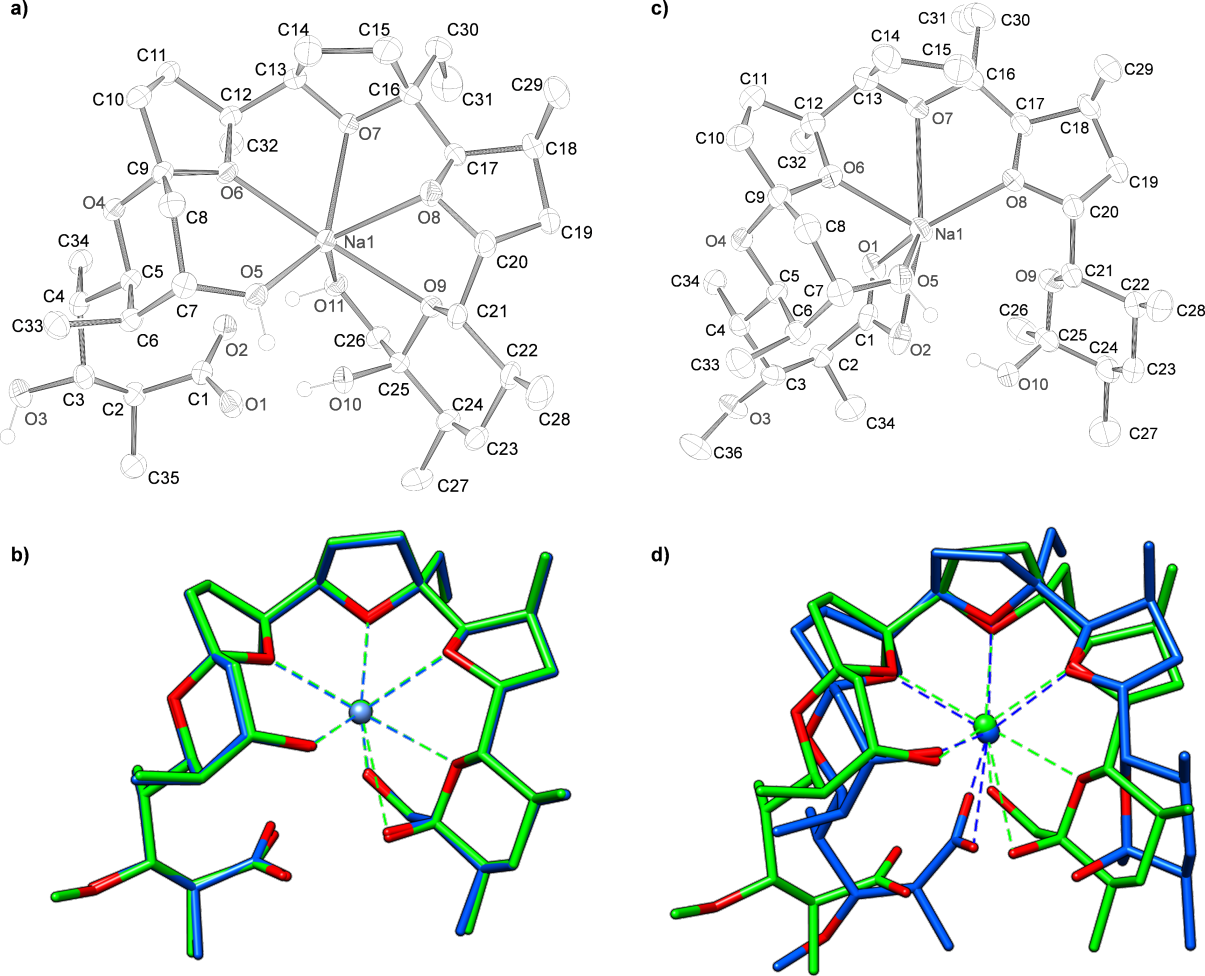
(a) Crystal structure of sodium demethylmonensin A (**4**) (ellipsoid probability = 50%); (b) overlay of **4** (blue) with the structure of sodium monensin A (**1,** green), oxygen atoms are coloured red); (c) crystal structure of sodium dehydroxymonensin A (**5**) and (d) the overlay of **5** (blue) with sodium monensin (**1**) (green).

To gain further insight into the metal-binding properties of demethylmonensin A and dehydroxymonensin A, they were each crystallised as their monosodium salts from an isohexane/ethyl acetate mixture and single crystals were subjected to X-ray structural analysis (Figure 2). Dehydroxydemethylmonensin A did not form crystals under any conditions tested. When the demethylmonensin A structure is overlaid on the structure of the monosodium complex of monensin A [26–29] the conformations of these complexes are revealed to be almost identical (Figure 2b). The obvious difference is that the hydroxy group at C-3 in demethylmonensin A lies on the otherwise wholly non-polar external surface of the complex, a feature that likely accounts for its lower effectiveness as an ionophore antibiotic [7]. Otherwise, both show a highly rigid structure in which the sodium cation is enveloped by the polyether and coordinated by four oxygen atoms in ether rings (O-6, O-7, O-8 and O-9), and by the oxygen atoms (O-5 and O-11, respectively) of the hydroxy groups at C-7 and C-26 (Figure 2a). The oxygen atoms of the carboxylic acid form two hydrogen bonds to, respectively, the oxygen atoms O-10 and O-11 at the other end of the carbon chain (Figure 2a), locking the conformation of the ligand into place around the cation. The importance of such hydrogen bonds for the stability of the complex is underlined by the conservation of this feature in the crystal structures of the polyether ionophores nigericin [30–31] and dianemycin (nanchangmycin) [32], where a carboxylic acid oxygen atom likewise form hydrogen bonds to the distal hydroxy groups equivalent to positions O-10 and O-11 in monensin A. Unlike monensin, these larger polyethers also show direct coordination of a carboxylic acid atom to the cation, as does grisoxin (30-dehydroxynigericin) in which the equivalent of the O-11 atom in monensin A is missing [33]. These differences have been ascribed to the increasing flexibility of the ligand in the larger polyethers, which also permits them to coordinate a wider selection of cations [34].

The structure of 26-dehydroxymonensin A complexed to a sodium ion proved to be remarkably different to that of monensin A (Figure 2a and 2d).

Here, both carboxylic oxygen atoms bind directly to the sodium ion, and the other end of the polyether (C-21–C-28) is fully released from coordination. Apparently, the loss of the H-bonds between the carboxylate (C-1) oxygen atom and the C-26 hydroxy group significantly compromises the integrity of the polyether shell surrounding the sodium ion. It has previously been shown that chemical modifications at the C-25 and/or C-26 hydroxy groups of monensin A lower both cation binding and antibiotic activity [35–36], presumably by the same mechanism. However, not all derivatives at C-26 behave in the same way: both natural and semisynthetic urethane derivatives of monensin A show maintained or even improved antibacterial activity [37–39]. The recently-determined crystal structure of the C-26-*O*-phenylurethane of monensin A sodium salt shows that although one of the carboxylate oxygen atoms coordinates the cation, this atom is also engaged in hydrogen bonding to the secondary alcohol borne at C-25, while the other carboxylate atom is engaged in an H-bond to the NH group of the urethane, thus maintaining the stability of the pseudocyclic structure [39]. The very different metal coordination pattern seen in dehydroxymonensin A and demethylmonensin A, although it highlights the importance of the C-26 *O*-hydroxylation for the integrity of the ionophore structure, is not directly relevant to the (evolution of) the biosynthetic pathway. As discussed above, the final steps leading to formation of the polyether rings take place with the polyketide in thioester linkage to a discrete acyl carrier protein (MonACPX) [20], and therefore the terminal carboxylate is not available as an alternative ligand for the bound cation until all the tailoring steps have been accomplished and the mature antibiotic is released by thioesterase action. It remains to be established whether the sodium cation is recruited into the ACP-bound polyether even before monensin is released.

Meanwhile, the convenient production of large amounts of advanced monensin-related metabolites by an engineered industrial strain now opens the way to the synthesis of the ACPX-thioesters of these compounds and a detailed study of the final steps of monensin biosynthesis using authentic substrates and recombinant MonD, MonE and MonCII [40–41].

## Experimental

**Construction of deletion strains:** For generation of the deletion mutants *ΔmonE*, *ΔmonD* and *ΔmonDΔmonE* of the *S. cinnamonensis* strain A519 [19] Redirect^©^-PCR-targeting technology was used according to the supplier's instructions [21,42]. The disruption cassettes were amplified from a *Hin*DIII-*Eco*RI-fragment of pIJ773 with the following primers (5' to 3') *monD*: oD1for: CGGCCGCCACATTCCCCGACCTGGT CGACCCGTCGTTCTAATTCCGGGGATCCGTCGACC; oD2rev:TCGTGAGCGCACATGCGCAGAGGGAAGCGTATGTGCTCATGTAGGCTGGAGCTGCTTC. *monE:* oE1for: CCCTT GTGCTGGGGGAGGAAAGCGGGGGCGGCACGCTCAATTCCGGGGCCGTCGACC; oE2rev: GTGACGAAGAACGCGACG ACCAAGAAGCCCCCGACGGTGTGTAGGCTGGAGCTGCTC. The DNA cassette used for reinsertion of *oriT* into the cosmid backbone: SCAT1for: CGCGAATTCTCATGTTTGACCGCTTA TCATCGATAAGCTTTCCCGCCAGCCTCGCAGAG; SCAT2rev: CACCGGAAGGAGCTGACTGGGTTGAAGGCTC TCAAGGGCGAAGTTCCTATACTTTCTA. The cosmids Cos2 (*monE*) and Cos11 (*monD*) from a cosmid library of *S. cinnamonensis* [16] were used as templates for PCR. All mutants were verified by sequencing on both forward and reverse strands with the following primers (5' to 3'): oE3rev: CGTTCCGTATGGAACGCGTCAAGAGCAGCTCC. oE4for: CGTCCGCGGGCCGGTACCTGACATTGAGG. oD3rev: CGC TCCAGGAGCTGCAGCGGGCCATACTCG. oD4for: GTCGCC CGTGCCGTGCCCGTGCGGTCGGTGGGCCTGACC. SCAT3for: CCTCGTACTGGAACGCCTCTC. SCAT4rev: CCTGTCCTACGAGTTGCATG. The subsequent sequencing revealed that the ΔmonD mutant contained an 82 bp “scar” instead of the expected in-frame 81 bp (traced to an error in one of the PCR primers) but since there is no downstream gene that might be subject to polar effects this mutant could still be used as though it were in-frame.

**Fermentation conditions:** Monensin (**1**) and the related metabolites **3**, **4** and **5** were produced as follows: 25 mL germination medium (soya flour (15.0 g L^−1^), dry yeast (1.5 g L^−1^), glucose (5 g L^−1^), dextrin (20 g L^−1^), CaCO_3_ (1 g L^−1^), pH 6.4) were inoculated with either *S. cinnamonensis* overproducer strain A519 or a mutant derived from it, and incubated for 48 h (30 °C, 140 rpm). Production medium (soya flour (32.6 g L^−1^), soya oil (3.0%), methyl oleate (1.0%), glucose (20.0 g L^−1^), Na_2_SO_4_ (2.2 g L^−1^), CaCO_3_ (2.0 g L^−1^), MnCl_2_·4H_2_O (330 mg L^−1^), FeSO_4_·7H_2_O (110 mg L^−1^), Al_2_(SO_4_)_3_·16H_2_O (705 mg L^−1^), K_2_HPO_4_·3H_2_O (75 mg L^−1^), MnCl_2_·4H_2_O (330 mg L^−1^), L-ascorbic acid (19 mg L^−1^), antifoam A (0.02%), pH 6.6–6.9), in portions of 50 mL (in 250 mL flasks) or 75 mL (in 500 mL flasks), was inoculated with the preculture (2.5 mL and 3.75 mL, respectively). The cultures were grown for 10 d at 30 °C and 120 rpm. Additional soya oil (3.0%) was supplemented on day 8.

**Product isolation and purification:** Polyether production was verified for each flask by TLC analysis of 0.5 mL samples of culture. For purification of polyethers, the 50 mL cultures of each mutant were extracted with ethyl acetate (3 × 100 mL). The combined organic layers were dried (MgSO_4_) and the solvent was evaporated, yielding 11–13 g of an oily residue. Most of the oil was separated by using a short silica column. A gradient (isohexane/ethyl acetate/isopropanol 20:20:(0–4) by volume) was used for elution, depending on the polarity of the ionophore. Triethylamine (0.1% by volume) was added to all eluents. The crude polyethers were obtained as brownish oils or solids. Further purification was performed by silica gel column chromatography (gradient: 1. isohexane/ethyl acetate 1:1 (0.1% NEt_3_), 2. isohexane/ethyl acetate/isopropanol 20:20:1 (0.1% NEt_3_), 3. ethyl acetate/isopropanol 20:1). For the less polar polyether **5** an additional gradient (isohexane/ethyl acetate 10:1 → 1:1) was used to separate side-products before elution with the above solvents.

**Analysis of monensin-related metabolites:** Monensin (**1**) and metabolites **3**, **4** and **5** were detected by TLC on silica gel as red spots after staining with (vanillin (6%), H_2_SO_4_ (1%) in ethanol). *R*_f_ values were ((isohexane/ethyl acetate/methanol 10:10:1)/acetic acid 80:1) **1**: 0.37, **3**: 0.29, **4**: 0.14, and **5**: 0.43. Monensin (**1**) and dehydroxymonensin (**5**) were quantified colorimetrically after vanillin staining (vanillin (3%), H_2_SO_4_ (0.5%), in methanol) [43]. Staining solution (100 µL) was added to 900 µL sample and incubated for 30 min at 55 °C. After cooling to room temperature, the absorption at 518 nm was measured and compared to reference samples. LC–MS: Crude extracts and purified compounds were analysed by ESI LC–MS/MS^2^/MS^3^ using a Phenomenex Prodigy 5 µm ODS3 100 Å column (250 × 4.6 mm) fitted to a Finnegan MAT LCQ mass spectrometer. The column was equilibrated in 20% 20 mM ammonium acetate buffer/80% methanol and compounds were eluted on a gradient to 100% methanol over 25 min. The mass spectrometer was set to record positive and negative ion scans between *m*/*z* 300 to 1000.

**Crystal structure data:** The crystal structures have been deposited at the Cambridge Crystallographic Data Centre and allocated the deposition numbers CCDC-793152(sp0601) for **4** and CCDC-793153(sp0602) for **5**. These crystallographic data can be obtained free of charge at http://www.ccdc.cam.ac.uk.

Sodium demethylmonensin A (**4**) crystallised from ethyl acetate/hexane as colourless needles, C_35_H_59_NaO_11_ (*M*_r_ = 678.83), empirical formula: C_36_H_64_NaO_10_·2H_2_O (*M*_r_ = 714.82), crystal size 0.23 × 0.18 × 0.12 mm^3^, orthorhombic, *a* = 8.36180(10) Å, *b* = 21.0283(3) Å, *c* = 21.1481(3) Å, *V* = 3718.56(9) Å^3^, *Z* = 4, ρ_calcd_ = 1.1277 Mg m^−3^, space group *P*2_1_2_1_2_1_, Mo Kα radiation (λ = 0.71073 Å), μ(Mo_Kα_) = 0.105 mm^−1^, F(000) = 1552. Sodium dehydroxy-monensin A (**5**) crystallised from ethyl acetate/hexane as colourless needles, C_36_H_61_NaO_10_ (*M*_r_ = 676.85), empirical formula: C_36_H_64_NaO_10_·1.5H_2_O (*M*_r_ = 703.87, solvent estimated), crystal size 0.46 × 0.42 × 0.35 mm^3^, orthorhombic, *a* = 8.04150(10) Å, *b* = 21.3058(3) Å, *c* = 24.1816(3) Å, *V* = 4143.05(9) Å^3^, *Z* = 4, ρ_calcd_ = 1.128 Mg m^−3^, space group *P*2_1_2_1_2_1_, Mo Kα radiation (λ = 0.71073 Å), μ(Mo_Kα_) = 0.091 mm^−1^, F(000) = 1532.
